# Effects of Wall-broken yeast powder on gut microbiota and serum metabolome in captive forest musk deer (*Moschus bere-zovskii*)

**DOI:** 10.3389/fvets.2026.1978027

**Published:** 2026-10-22

**Authors:** Yongjiang Wu, Tianjun Chen, Zaixiang Ying, Shan Xie, Wei Su, Ningqian Tan, Mingcheng Cai, Dejun Zeng, Chenglu Zhang, Qiuping Huang, Siqi Peng, Zhonggui Jiang, Qinlin Yang

**Affiliations:** 1 College of Smart Agriculture, Chongqing University of Arts and Sciences, Chongqing, China; 2 Chongqing Institute of Medicinal Plant Cultivation, Chongqing University of Chinese Medicine, Chongqing, China; 3 Department of Ophthalmology, Yongchuan Hospital of Chongqing Medical University, Chongqing, China

**Keywords:** forest musk deer, gut microbia, immunity, serum metabolisme, wall-broken yeast

## Abstract

Forest musk deer (*Moschus berezovskii*), a nationally protected species, has experienced a rapid population decline, highlighting the need to improve captive breeding strategies. Captive individuals are more susceptible to gastrointestinal disorders than wild individuals, making dietary regulation important for intestinal health. Wall-broken yeast (BWY) powder, has attracted increasing attention as a functional feed additive due to its potential roles in regulating intestinal health, microbial composition, and host metabolism. This study evaluated the effects of wall-broken yeast powder on gut microbiota, and serum metabolome in forest musk deer. Fourteen healthy male forest musk deer aged 4.5 years, were randomly assigned to two groups (*n* = 7). The CK group was fed a basal diet, whereas the BWY group was fed the basal diet supplemented with 15 g/kg DM wall-broken yeast powder for a 7 d adaptation period and a 30 d experimental period. 16S rDNA sequencing showed that BWY supplementation altered gut microbial **c**ommunity structure based on β-diversity analysis, with *Ruminobacter*, UCG-005, and *Bacteroides* showing increased relative abundance in the BWY group. Serum metabolomics identified 69 differential metabolites between the CK and BWY groups (VIP > 1 and *P* < 0.05), with upregulated metabolites mainly enriched in amino acids and their metabolites, hormones and hormone-related substances, and glycerophospholipids. Key metabolic pathways included ABC transporters, glycerophospholipid metabolism, phenylalanine metabolism, and β-alanine metabolism. Correlation analysis showed that *Prevotellaceae_UCG-004* was positively associated with serum salidroside levels, whereas *Ruminobacter* was positively correlated with quinolinic acid, Lys-Gly, Gly-Lys, and Cyclo(Pro-Leu) levels (*P* < 0.05). Overall, wall-broken yeast powder supplementation may modulate gut microbial composition and affect amino acid metabolism in forest musk deer.

## 1 Introduction

Since the 1950s, habitat degradation and overexploitation have led to a sharp decline in the population of forest musk deer (*Moschus berezovskii*, FMD) in Southwest China, and FMD has been listed as a nationally protected species (1, 2). FMD is a small forest-dwelling ruminant that secretes musk, a highly valuable medicinal product. Consequently, the supply of musk is insufficient to meet market demand. Moreover, FMD individuals usually live alone or in pairs and have specific habitat preferences, which poses challenges for captive breeding. In 1958, China began the domestication and breeding of FMD to increase musk production and release captive-bred deer. However, captive forest musk deer are more susceptible to disease than wild individuals and frequently develop gastrointestinal diseases, including diarrhea, dyspepsia, gastroenteritis, and gastrointestinal hemorrhage, as well as pneumonia, uterine inflammation, parasitosis, and urolithiasis. Gastrointestinal diseases are the most common, with a mortality rate of 30%, which is higher than that in wild forest musk deer and represents a major constraint to large-scale captive breeding (2–4). The microorganisms present in the gastrointestinal tract, referred to as the gut microbiota, begin to colonize animals after birth and play key roles in host metabolism, growth and development, and immune responses (5, 6). Maintaining microbial balance is therefore critical for intestinal health and disease resistance in ruminants (3, 5, 6). Diet is a key factor affecting animal intestinal microbiota. During the growth and development of FMD, improving the palatability and nutrient composition of feed is essential for improving health status, feed efficiency, and the scalability of captive breeding.

Wall-broken yeast (BWY) powder is a processed yeast in which the cell wall is disrupted to improve nutrient availability, allowing intracellular nutrients to be more readily released and utilized. This process may improve its application value compared with conventional yeast powder in animal feed. Previous studies have demonstrated that yeast cell wall products also have beneficial effects in other ruminants. Dietary supplementation with yeast and yeast cell wall polysaccharides improved growth performance, modulated rumen microbial populations, and reduced lipopolysaccharide production in beef cattle (7). In addition, yeast supplementation has been shown to regulate rumen microbial communities and alleviate rumen epithelial injury in sheep (8). The flavoring substances of yeast are derived from amino acids and peptides, such as glutamic acid, guanosine 5′-monophosphate (5′-GMP), and inosine-5′-monophosphate (5′-IMP), which may enhance feed palatability, improve digestive capacity, and thereby improve productive performance (9). 5′-IMP has been evaluated for its effects on growth performance in pigs, and Bonagurio et al. (10) found that dietary supplementation with 5′-IMP had positive effects on growth performance and carcass characteristics. Yeast is also rich in high-quality protein, which supports rumen microbial activity and overall productive performance (11–13). Previous studies in livestock have demonstrated that dietary yeast supplementation can improve animal health and production performance. Previous studies in livestock have demonstrated that dietary yeast supplementation can improve animal health and production performance. Yeast-derived products have been shown to enhance growth performance and regulate intestinal health and immune responses in pigs (14, 15), while yeast cell wall supplementation improved intestinal integrity and immune traits in poultry (16). In ruminants, yeast supplementation modulated rumen microbial communities and metabolic profiles, thereby contributing to improved gastrointestinal health (8). However, the effects of wall-broken yeast powder on gut microbiota and serum metabolism in captive forest musk deer remain unclear. In addition, yeast is a rich source of nucleic acids, particularly RNA, which accounts for a considerable proportion of yeast biomass. During digestion or processing, yeast-derived nucleic acids can be hydrolyzed into nucleotides and nucleosides, serving as important dietary nucleotide sources (17, 18). Dietary nucleotides have been reported to exert beneficial effects on physiological functions in both humans and animals, including supporting intestinal health and immune regulation (19). Nucleotide supplementation has been shown to promote gastrointestinal development and act as an immunomodulator (20, 21), including increased expression of immune-related proteins such as nuclear factor-kappa B (*NF-*κ*B*), interleukin-6 (*IL-6*), and tumor necrosis factor-alpha (*TNF-*α) in weaned piglets (14, 15). Trace elements are essential for growth and metabolism, and adequate dietary supplementation is required. Organic trace elements derived from yeast exhibit higher bioavailability. Chromium yeast increased plasma chromium levels in *Camelus dromedarius* pups and may alleviate hyperglycaemia in KK-AY mice, while selenium yeast improved productive performance and antioxidant capacity in livestock (22–25). In addition, yeast contains enzymes that enhance nutrient digestion and feed efficiency (26). The yeast cell wall consists mainly of polysaccharides and proteins, including β-glucan, mannose, and chitin (27). β-glucan shows a better immunomodulatory function (28) and can be recognized and processed by immune cells, including macrophages, natural killer (NK) cells, and dendritic cells, thereby enhancing host immune responses against pathogens and tumor cells (29, 30). Supplementation with yeast cell wall improves intestinal integrity and health in broilers (16). Collectively, these findings suggest that BWY supplementation may confer nutritional, immunological, and intestinal health benefits, thereby improving host health and metabolic function. However, the specific effects of BWY on gut microbiota and host metabolism remain poorly characterized in wild ungulates under captive conditions.

Therefore, this study investigates the effects of dietary BWY powder on gut microbiota composition and serum metabolite profiles, exploring the correlations between microbial shifts and metabolic changes in the forest musk deer (*Moschus berezovskii*).

## 2 Materials and methods

### 2.1 Experimental material

BWY powder was purchased from Xuzhou Saifu Biotechnology Co., Ltd. (Jiangsu, China). The BWY was a single ingredient wall broken yeast preparation without carrier additives. According to the manufacturer's quality inspection report, the BWY contained 42.20% crude protein, 26.39% polysaccharides, 7.07% moisture, and 7.13% ash, and exhibited a cell wall disruption rate of >90%. The experimental animals and facilities used in this study were provided by the Chongqing Institute of Medicinal Plant Cultivation (Chongqing, China). The institute is one of the earliest institutions in China to conduct research on the artificial domestication and breeding of FMD, it has over 10 years of experience in the captive breeding and management of FMD. During the experimental period, all FMD were subjected to systematic health monitoring conducted regularly by a specialized management team comprising licensed veterinarians and professionally trained animal care technicians. All experimental procedures involving forest musk deer were approved by the Animal Ethics Committee of Chongqing University of Arts and Sciences (approval number: CQWLDF033). All procedures were conducted in accordance with institutional guidelines for the care and use of experimental animals.

### 2.2 Experimental design

Fourteen male FMD with comparable health status (average age: 4.5 years and average body weight: 7.0 ± 0.3 kg) were randomly assigned to two groups, with 7 individuals in each group. Due to the limited availability of captive forest musk deer and ethical considerations for this protected species, the sample size was determined based on the available healthy individuals and previous studies involving captive FMD. The CK group was fed a standard diet without BWY supplementation, whereas the BWY group received the standard diet supplemented with BWY at 15 g/kg DM. According to the manufacturer's specification, BWY contained 42.20% crude protein and 26.39% polysaccharides. At the addition rate of 15 g/kg DM, this contributed approximately 0.65% to dietary crude protein. No nutritional compensation was applied in the control diet, as the resulting differences in energy and protein were within normal formulation variation (±3%−5%). Both diets were formulated to meet or exceed nutrient requirements for FMD. The inclusion level of BWY (15 g/kg DM) was selected based on preliminary feeding observations and previous studies demonstrating that moderate dietary supplementation of yeast-derived products can exert beneficial effects on rumen fermentation, intestinal microbial composition, host physiological responses, and metabolic functions in livestock species (31–33). The feeding amount per meal was maintained at (300.00 ± 10.00) g based on the daily nutritional requirements and feeding management practices of captive FMD. Fresh water was provided *ad libitum* throughout the experimental period. The composition and nutritional levels of the standard diet are shown in Table 1. Each FMD was housed in two separate enclosures, one designated for feeding the standard diet and the other for daily resting, the two experimental groups were maintained under identical conditions, each FMD was housed in an individual enclosure (4.8 m × 2.5 m) equipped with feeding and resting areas. During the experimental period, the temperature was maintained at 26.7–32.4 °C, and relative humidity was maintained at 48.68%−88.79% under natural photoperiod conditions. One week prior to the experiment, the enclosures were thoroughly cleaned and disinfected, and all relevant facilities and ventilation systems were inspected to ensure proper operation. During the experimental period, standard management protocols were strictly followed, and feeding was carried out daily at 3:00 p.m. The pretrial period lasted 7 d, followed by a formal trial period of 30 d. The 7 d pre-trial period was designed to allow animals to adapt to the experimental diet and housing conditions and to minimize the influence of environmental stress on subsequent measurements.

**Table 1:** Composition and nutrient levels of the basal diet (DM basis, excluding BWY supplementation) ^c^ %.

Items	Content
Ingredients	
Leaves of *Pittosporum truncatum Pritz*	50.00
Corn	30.00
Soybean meal	11.80
wheat bran	3.50
Corn germ meal	2.40
NaCl	0.30
CaHPO4	0.50
Limestone	0.50
Premix^a^	1.00
Total	100.00
Nutrient levels	
Dry matter	73.50
Metabolic energy/(MJ /kg)^b^	8.24
Crude protein	10.64
Neutral detergent fiber	30.23
Acid detergent fiber	15.26
Calcium	0.57
Phosphorus	0.31

^a^The premix provided the following per kg of the basal ration: VA 7000 IU, VD 1800 IU, VE 40 IU, Cu 12 mg, Fe 60 mg, Mn 50 mg, Zn 40 mg, I 1.0 mg, Se 0.27 mg, Co 0.3mg.

^b^Metabolic energy is derived from calculations, whereas the others are obtained through measurements

^c^BWY was supplemented on top of the basal diet at 15 g/kg DM and was not included in the calculation of the basal diet composition.

### 2.3 Sample collection

Considering that FMD are highly sensitive to changes in the external environment, on the final day of the experiment (day 30), blood sampling was conducted in three randomly selected FMD from each group. Venous blood was collected from the hindlimb saphenous vein into clot activator tubes. After centrifugation at 3,000 rpm for 10 min at 4 °C, the separated serum was stored at −80 °C for subsequent serum metabolomics analysis. Subsequently, fecal samples were collected from all FMD individuals immediately after natural defecation. Freshly voided samples were aseptically collected using sterile disposable gloves and transferred into sterile sampling tubes. Samples were immediately frozen in liquid nitrogen and stored at −80 °C until 16S rDNA sequencing analysis.

### 2.4 Fecal microbial DNA extraction and 16S rDNA sequencing

Approximately 200 mg of fecal sample was used for total microbial DNA extraction using the HiPure Stool DNA Kit (Guangzhou Magen Biotechnology Co., Ltd., China). The concentration and purity of DNA were determined using a NanoDrop 2,000 spectrophotometer (Ther-moFisher Scientific, Inc., USA). The V3–V4 hypervariable region of the bacterial 16S rDNA gene was amplified using the universal primers 338F 5′-ACTCCTACGGGAGGCAGCAG3′) and 806R 5′-GGACTACHVGGGTWTCTAAT3′). An 8-bp barcode sequence was added to the5′ end of each primer to enable sample identification. PCR amplification was performed using an ABI 9,700 thermal cycler (Applied Biosystems, Inc., USA). The amplified products were purified and quantified, and then high-throughput sequencing was performed using the Illumina NovaSeq 6,000 sequencing platform (Illumina, Inc., USA).

Negative controls were included during DNA extraction and sequencing procedures to monitor potential contamination. Raw sequencing reads were screened and filtered to remove low-quality sequences, chimeric sequences, and potential contaminant sequences before downstream analysis. Raw sequencing data were quality-filtered and merged using PEAR software (v0.9.6) (34). The assembled sequences were processed using the Vsearch software (v2.7.1) to remove chimeric sequences and short sequences shorter than 120 bp (35). High-quality sequences were clustered into operational taxonomic units (OTUs) at a 97% similarity threshold using the UPARSE algorithm in Vsearch software (2.7.1) (36). OTU representative sequences were aligned against the Silva 138, Unite 8.2, and GenBank nonredundant nucleotide (NT) databases using the BLAST algorithm to obtain taxonomic annotation information with an e-value threshold of ≤ 1 × 10^−5^ (37, 38). Alpha and beta diversity indices were calculated using QIIME software (v1.8.0) (39). Rarefaction curves were generated using QIIME software (v1.8.0) to evaluate sequencing depth and sampling completeness of microbial communities. Beta diversity was assessed using Bray–Curtis similarity and UniFrac distance (40), and the results were visualized with R software (v3.6.0). The differences in microbial community composition between groups were evaluated using ANOSIM analysis with 999 permutations. LEfSe analysis was performed using Python (v2.7) (41).

### 2.5 Serum metabolomics analysis

Following the gut microbiota analysis, serum samples were subjected to metabolomics analysis to further investigate the metabolic changes associated with BWY supplementation. Samples were removed from the −80 °C freezer and thawed slowly on ice, followed by vortex mixing. A 50 μL serum sample was mixed with 300 μL of extraction solvent containing an internal standard (ACN: Methanol = 1:4, V/V), vortexed for approximately 3 min. The mixture was centrifuged at 12,000 rpm for 10 min at 4 °C, and 200 μL of the supernatant was transferred into a new labeled centrifuge tube and incubated at −20 °C for 30 min. The samples were then centrifuged again at 12,000 rpm for 3 min at 4 °C, and 180 μL of the supernatant was transferred into the corresponding sample vial for LC–MS analysis. Serum metabolite relative contents were determined using an LC ESI MS/MS system (UPLC, ExionLC AD, https://sciex.com.cn/; MS, QTRAP^®^ System, https://sciex.com/). Samples were separated using an Agilent 1,290 Infinity LC ultra-high-performance liquid chromatography system (UHPLC) equipped with a HILIC column. The column temperature was maintained at 25 °C, with a flow rate of 0.5 mL/min and an injection volume of 2 μL. The mobile phases consisted of solvent A (water containing 25 mM ammonium acetate and 25 mM ammonia solution) and solvent B (acetonitrile). The gradient elution program was as follows: 0–0.5 min, 95% B; 0.5–7 min, solvent B linearly decreased from 95% to 65%; 7–8 min, solvent B linearly decreased from 65% to 40%; 8–9 min, solvent B was maintained at 40%; 9–9.1 min, solvent B linearly increased from 40% to 95%; and 9.1–12 min, solvent B was maintained at 95%. Throughout the analysis, samples were maintained at 4 °C in an autosampler. To minimize the effects of signal fluctuations during instrument detection, all samples were continuously analyzed in a randomized order. Raw data were imported into Progenesis QI (version 3.0) for peak extraction, alignment, and normalization, and metabolite annotation was performed based on the HMDB and Metlin databases.

Statistical analyses were performed in R(www.rproject.org). Unsupervised principal component analysis (PCA) was conducted using the prcomp function, Com-plexHeatmap package was used for the visualization of hierarchical cluster analysis (HCA), and the cor function was applied to calculate the Pearson correlation coefficient (PCC), MetaboAnalystR (version 3.0) was used for the screening of differential metabolites; partial least squares discriminant analysis (PLS-DA) was also performed. Differential metabolites were identified based on VIP >1 and *p* < 0.05 (Student's t-test). For gut microbiota analysis, taxa with significant differences between groups were identified using LEfSe analysis with an LDA score threshold of >2.0. Volcano plots were generated based on these criteria to visualize significantly altered metabolites, with fold change values used to indicate the direction and magnitude of metabolite changes rather than as an independent screening threshold. Metabolites with fold change values greater than 1.0 were considered upregulated, whereas those with fold change values lower than 1.0 were considered downregulated in the BWY group compared with the CK group. The identified metabolites were annotated using the KEGG Compound data-base(http://www.kegg.jp/kegg/compound/) and mapped to the KEGG Pathway data-base(http://www.kegg.jp/kegg/pathway.html) for pathway analysis. These analyses were performed by Beijing Ai'erwei Gene Technology Co., Ltd.

### 2.6 Correlation analysis for differential microbes and metabolites

Correlation analysis was performed between differential microbiota and metabolites. Spearman correlation coefficients between the gut microbiota and metabolites were calculated using the psych package in R, and the correlation results were visualized using the heatmap package.

### 2.7 Statistical analysis

All data were analyzed using an independent samples *T*-test. Results are presented as mean ± standard deviation (SD), and differences were considered statistically significant at *p* < 0.05. Microbial functions were predicted using PICRUSt2 (version 2.1.4), and significantly differential metabolites were identified using the *T*-test. Statistical analyses were performed using SPSS software (version 27.0.1).

## 3 Results

### 3.1 Gut microbiome 16S rDNA sequencing analysis

#### 3.1.1 The effects of wall-broken yeast powder on gut microbiota in FMD

At >97% similarity, the CK and BWY groups shared 2,866 operational taxonomic units (OTUs), whereas 987 and 1,097 OTUs were unique to the CK and BWY groups, respectively (Figure 1a). Rarefaction curve analysis was performed to evaluate whether the sequencing depth was sufficient to characterize the fecal microbial communities. As shown in Supplementary Figure S1, the OTU numbers increased rapidly at the initial sequencing stage and gradually approached a plateau with increasing sequencing reads in all samples. These results indicate that the sequencing depth was sufficient to capture the majority of bacterial diversity in the fecal microbiota of FMD. The Chao1, observed species, PD whole tree, and Shannon indices were lower in the BWY group than in the CK group, although the differences were not significant (*P* > 0.05; Figures 1c–f). Beta diversity analysis based on OTU profiles showed a similar microbial community structure between the CK and BWY groups. ANOSIM analysis based on Bray–Curtis distance revealed no significant differences in overall microbial community composition between groups (*R* = −0.0214, *P* = 0.538; Figure 1b).

**Figure 1:**
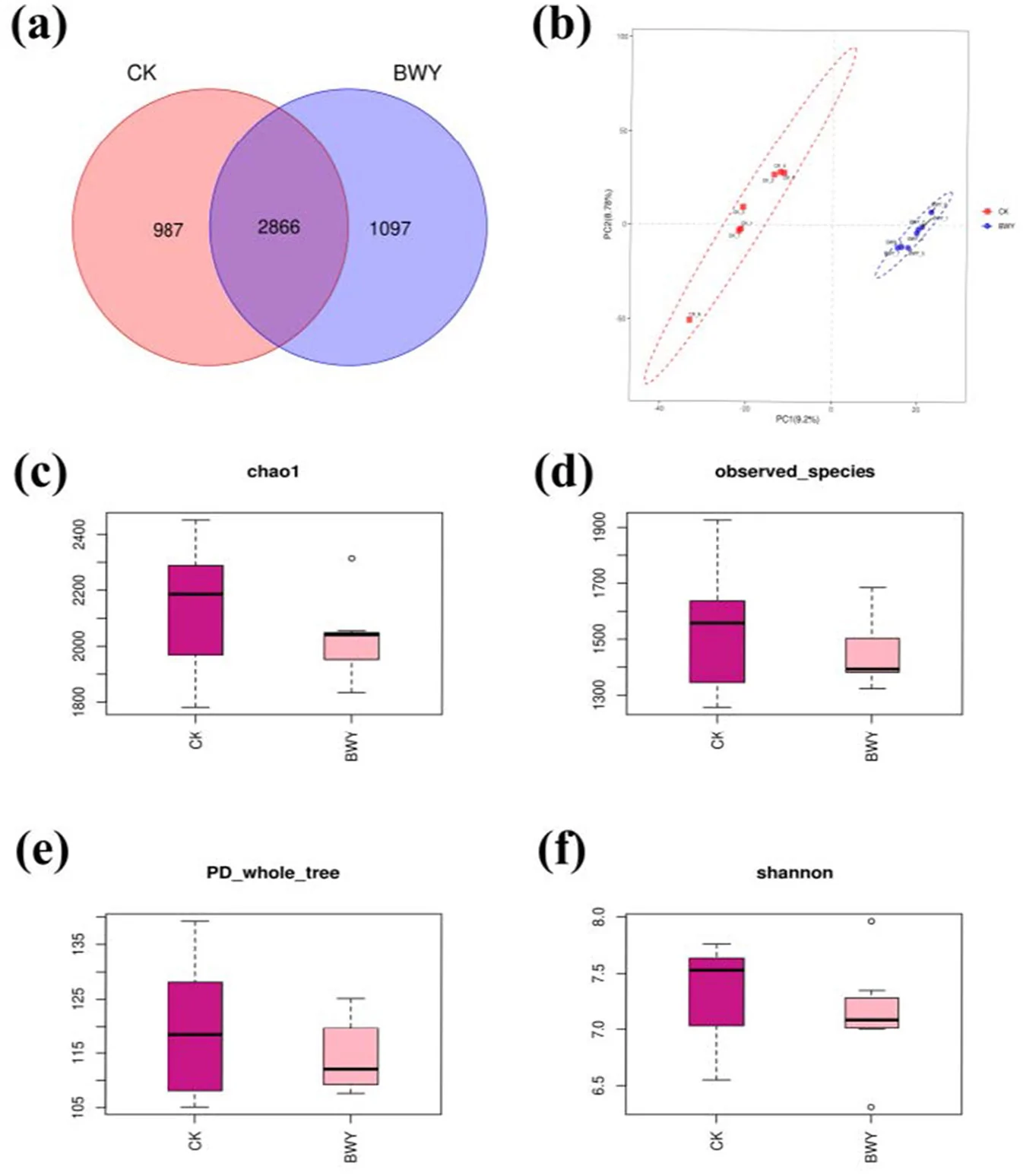
Effect of wall-broken yeast powder on gut microbial diversity. CK, Standard feed group. BWY, Wall-Broken Yeast powder group. Venn diagram representing the number of unique and shared OTUs in the two groups **(a)**. PLS-DA analysis based on OTU profiles. Beta-diversity differences between CK and BWY groups were further evaluated using ANOSIM analysis based on Bray–Curtis distance **(b)**. Box plot of Alpha Diversity Index(Chao1, Observed_species, PD_whole_tree and Shannon) **(c–f)**.

#### 3.1.2 Analysis of the composition of the gut microbiota

At the phylum level, Firmicutes, Bacteroidota, and Proteobacteria were the dominant phyla in FMD (Figure 2a). Compared with the CK group, the BWY group showed an increased relative abundance of Firmicutes and a decreased relative abundance of Bacteroidota, whereas Proteobacteria did not change significantly. At the genus level, the dominant taxa were *Ruminobacter*, UCG-005, and *Bacteroides* (Figure 2b), all of which showed increased relative abundance in the BWY group.

**Figure 2:**
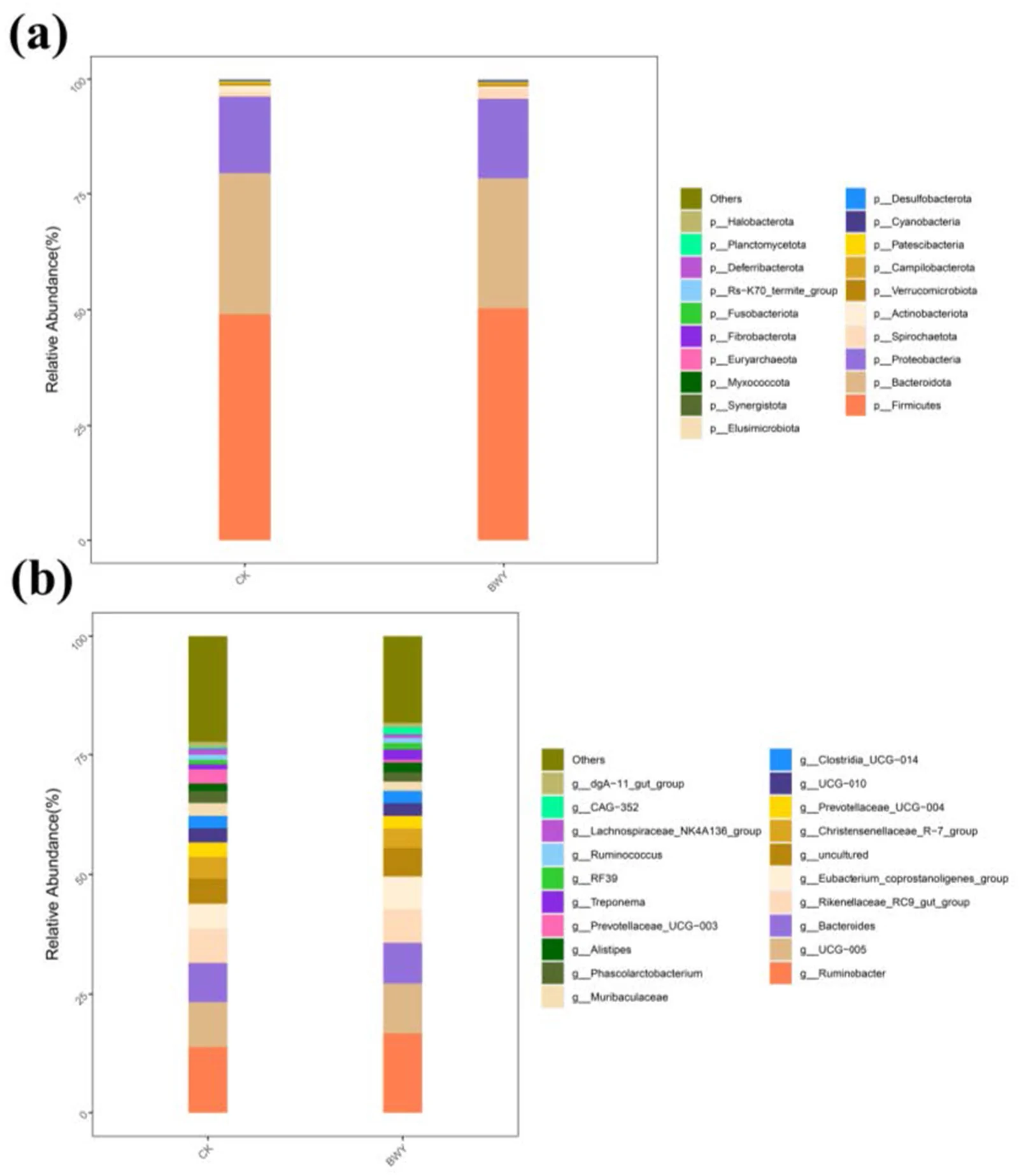
Effect of wall-broken yeast powder on gut microbial composition. **(a)** Gut Microbial Composition (Phylum Level). **(b)** Gut Microbial Composition (Genus Level).

#### 3.1.3 Identification of differentially abundant gut microbial taxa

Linear discriminant analysis effect size (LEfSe) was used to identify taxa with significantly different relative abundances between groups. In the LEfSe cladogram, five taxa and one lineage were differentially enriched between the CK and BWY groups (Figure 3a). The BWY group was characterized by enrichment of f__Barnesiellaceae and s__bacterium_NLAE_z1_H40, whereas the CK group showed enrichment of p__Actinobacteriota, c__Actinobacteria, f__Corynebacteriaceae, and g__Corynebacterium. The Actinobacteriota lineage showed the greatest discriminatory contribution according to LDA analysis (Figure 3b).

**Figure 3:**
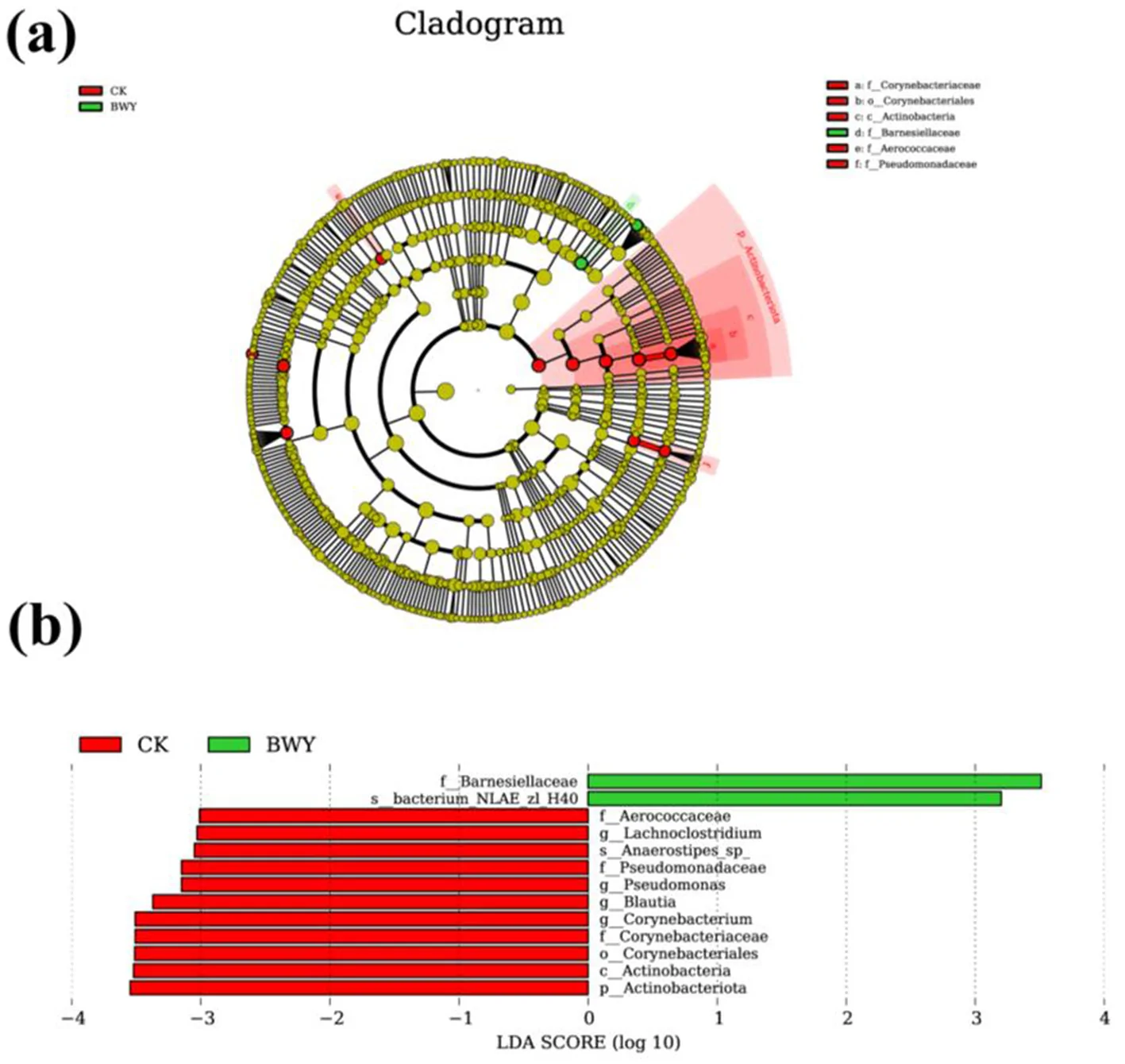
LEfSe analysis of differential microbial taxa between CK and BWY groups. **(a)** Cladogram showing significantly different taxa identified by LEfSe analysis. **(b)** Linear discriminant analysis (LDA) score distribution of differential taxa. Red indicates taxa enriched in the CK group, and green indicates taxa enriched in the BWY group.

#### 3.1.4 Predictive analysis of gut microbial function

Functional prediction analysis revealed significant differences in gut microbial metabolic pathways between the CK and BWY groups (Figure 4). Compared with the CK group, the BWY group showed enrichment of D-glutamine and D-glutamate metabolism, ribosome, cell cycle-Caulobacter, protein export, and homologous recombination pathways.

**Figure 4:**
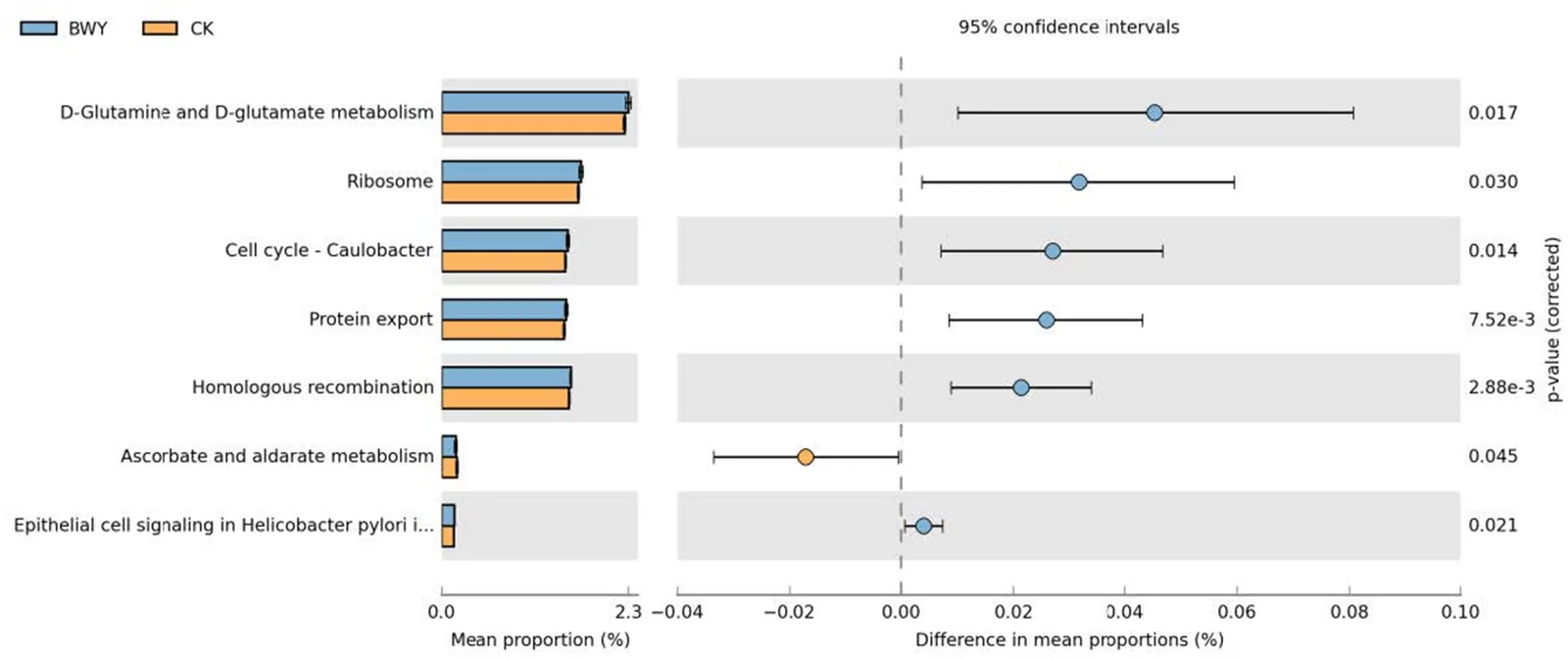
Differential analysis of functional prediction of gut microbiota in CK and BWY groups. CK, Standard feed group. BWY, Wall-Broken Yeast Powder Group.

### 3.2 Serum-wide targeted metabolomics analysis

#### 3.2.1 Serum metabolite multivariate statistical analysis

Based on targeted serum metabolomics analysis, the effect of BWY intervention on the serum metabolic profile of FMD was investigated using multivariate statistical analysis. Orthogonal partial least squares discriminant analysis (OPLS-DA) was performed to evaluate the separation of serum metabolic profiles between the CK and BWY groups and to establish the OPLS-DA model (Figure 5a). The model was evaluated using R^2^Y, which reflects explanatory ability, and Q^2^, which represents predictive ability. Higher R^2^Y and Q^2^ values indicate better model explanatory and predictive performance. As shown in Figure 5a, the serum metabolic profiles of the CK and BWY groups were clearly separated, suggesting that BWY intervention induced marked changes in the serum metabolic profile of FMD. Moreover, as shown in Figure 5b, the R^2^Y and Q^2^ values were 0.999 and 0.65, respectively, indicating that the OPLS-DA model showed good explanatory and predictive performance.

**Figure 5:**
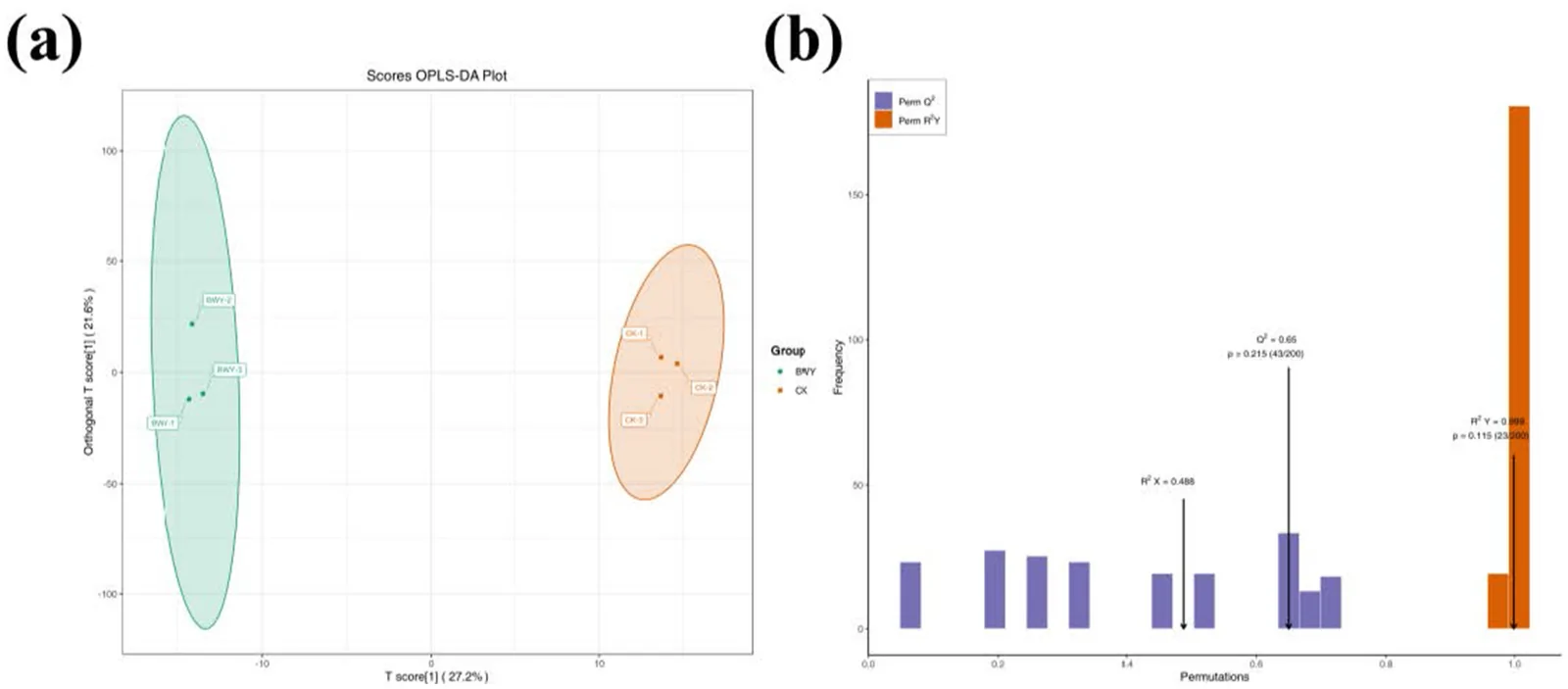
OPLS-DA analysis of serum metabolic profiles between CK and BWY groups. **(a)** OPLS-DA score plot showing the separation between CK and BWY groups. Yellow and green dots represent CK and BWY samples, respectively. **(b)** Validation results of the OPLS-DA model based on permutation testing.

#### 3.2.2 Serum differential metabolites screening

Differential metabolites were identified based on VIP ≥ 1 in the OPLS-DA model and *P* ≤ 0.05 in the *t*-test. Fold change values were calculated to describe the magnitude and direction of metabolite changes, but no additional fold change threshold was applied for differential metabolite screening. The identified metabolites were visualized using a volcano plot (Figure 6a). A total of 69 differential metabolites were identified between the CK and BWY groups, including 63 upregulated metabolites and 6 downregulated metabolites. In the volcano plot, red and green indicate upregulated and downregulated metabolites, respectively. The detailed information of these metabolites, including metabolite annotation, VIP values, *P* values, fold changes, and regulation patterns, is provided in Supplementary Table S1. Hierarchical clustering analysis further showed distinct clustering patterns of these differential metabolites, with red and green indicating relatively high and low abundance, respectively (Figure 6b). As shown in Table 2, compared with the CK group, metabolites such as estrone, galacturonic acid, histidine, and L-glutamic acid were upregulated in the BWY group, whereas Phe-Lys, FFA (22:4), and LPA (18:1/0:0) were downregulated. In addition, salidroside was significantly decreased in the BWY group compared with the CK group. These results indicate that BWY supplementation significantly modulates serum metabolic profiles in FMD.

**Figure 6:**
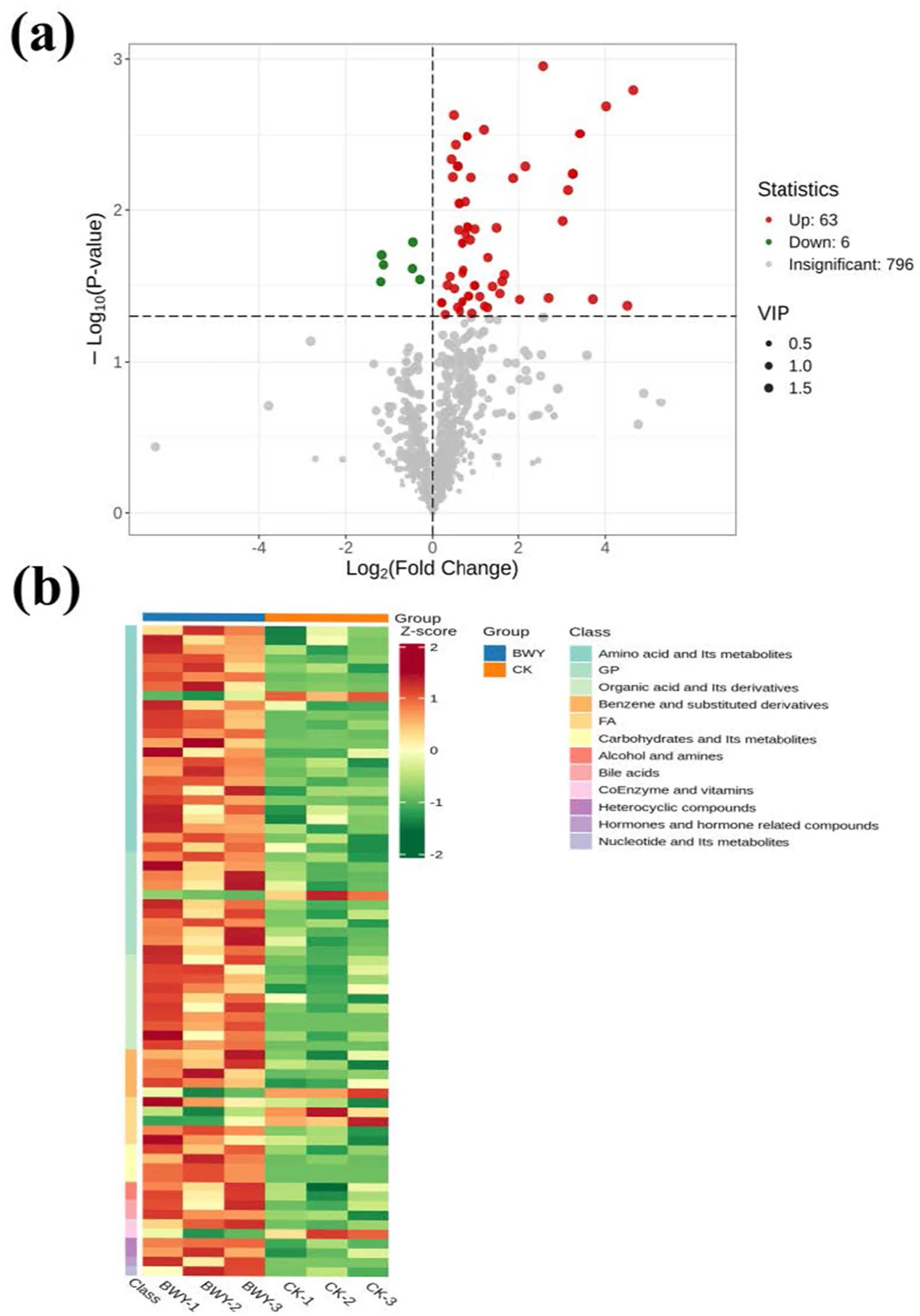
Volcano plot of differential metabolites in the CK and BWY groups **(a)**. The size of the dots represents the VIP value, where green dots represent the down-regulation of the significant difference and red dots represent the up-regulation of the significant difference. Clustering heatmap of differential metabolites in the CK and BWY groups **(b)**. CK, Standard feed group. BWY, Wall-Broken Yeast powder group.

**Table 2:** Representative differential metabolites identified between CK and BWY groups.

Items	VIP	*P*-value	Fold change	Trend
LPC(0:0/18:1)	1.82	< 0.01	1.74	up
Pro-Ile	1.88	< 0.01	25.06	up
Cyclo(Pro-Val)	1.86	0.04	22.78	up
Phe-Lys	1.75	0.02	0.73	down
Estrone	1.82	< 0.01	1.74	up
Cyclo(Pro-Leu)	1.88	< 0.01	25.06	up
Glu-His	1.86	0.04	22.78	up
LPC(19:2/0:0)	1.82	0.03	3.20	up
Piperidine acid	1.87	< 0.01	1.40	up
Quinolinic acid	1.86	< 0.01	1.38	up
Galacturonic acid	1.80	< 0.01	1.45	up
Deoxycholic acid	1.76	< 0.01	1.88	up
Histidine	1.85	< 0.01	1.30	up
Traumatic acid	1.68	0.04	2.98	up
FFA(22:4)	1.67	0.02	0.72	down
LPA(18:1/0:0)	1.86	0.02	0.40	down
Salidroside	1.61	0.03	0.44	down
Lys-Gly	1.58	0.04	1.16	up
L-Glutamic Acid	1.79	< 0.01	1.53	up
1,5-Anhydro-D-Glucitol	1.92	< 0.01	10.70	up

Metabolites were classified as upregulated or downregulated according to their relative abundance changes in the BWY group compared with the CK group. Metabolites with fold change >1.0 were classified as upregulated, whereas metabolites with fold change < 1.0 were classified as downregulated.

#### 3.2.3 Serum metabolic pathway analysis

Based on KEGG enrichment and topology analysis. As shown in Figure 7a, the annotated pathways were classified into six KEGG categories, including metabolism, genetic information processing, environmental information processing, cellular processes, organismal systems, and human diseases. Among these pathways, metabolic pathways, glycerophospholipid metabolism, ABC transporters, phenylalanine metabolism, carbon metabolism, and β-alanine metabolism showed major enrichment differences between groups (Figure 7b). These results suggest that BWY supplementation may regulate lipid metabolism and other metabolic pathways in FMD.

**Figure 7:**
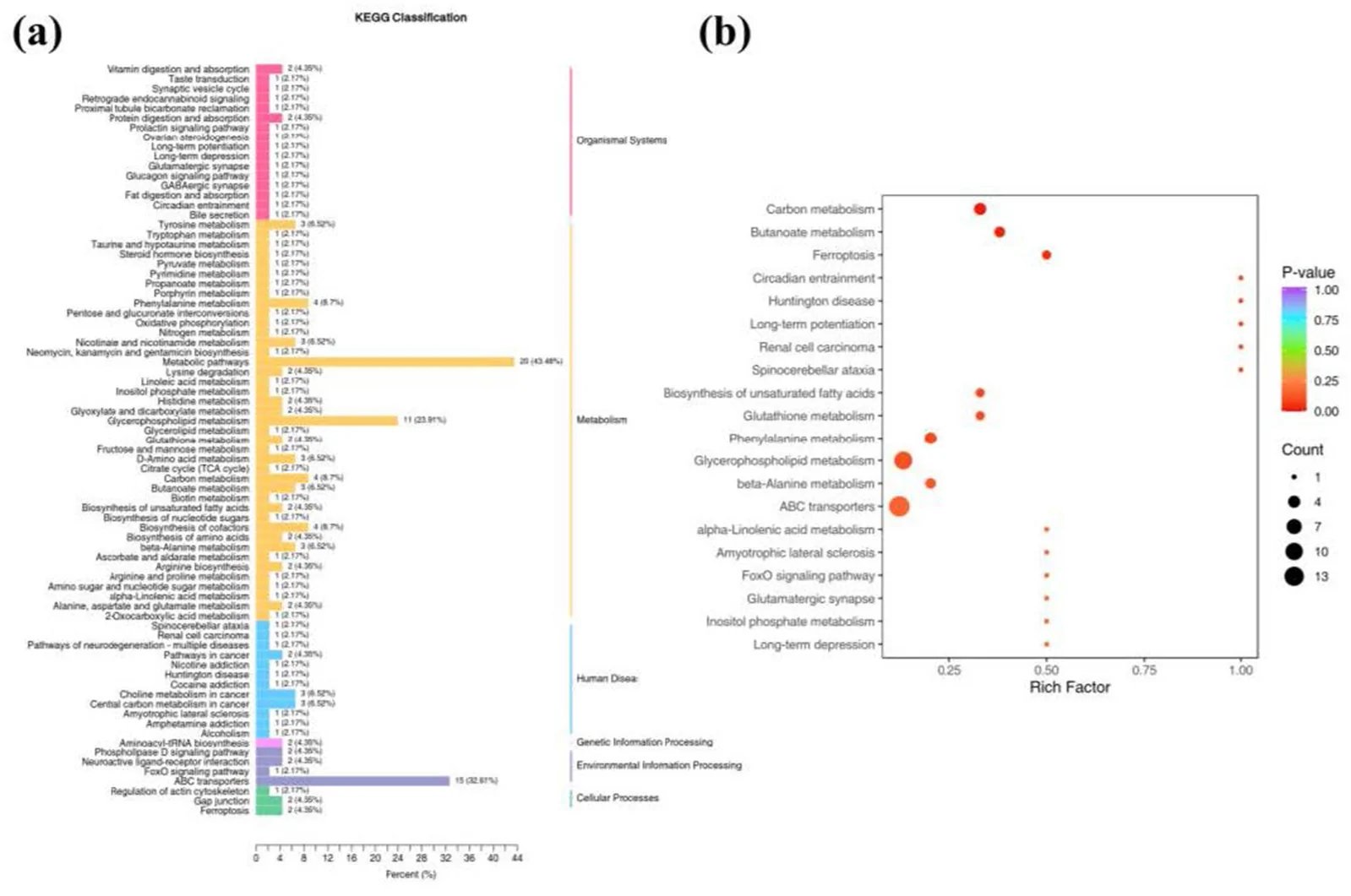
Differential metabolite pathway classification plot **(a)**, differential metabolite pathway enrichment plot **(b)**. larger Rich Factor values indicate greater enrichment, and the color of the dots reflects the size of the *P*-value, with redder indicating more significant enrichment. CK, Standard feed group. BWY, Wall-Broken Yeast powder group.

### 3.3 Correlation analysis for Ddfferential microbes and metabolites

Pearson correlation analysis was performed between differential serum metabolites meeting the criteria of VIP >1 and *P* < 0.05 and the differential taxa identified by LEfSe analysis. As shown in Figure 8, the relative abundance of *Prevotellaceae_UCG-004* was significantly and positively correlated with serum salidroside content. The relative abundance of *Ruminobacter* was significantly and positively correlated with serum quinolinic acid, Lys-Gly, Gly-Lys, and Cyclo(Pro-Leu) levels. In addition, the relative abundance of *Prevotellaceae_UCG-004* was significantly correlated with serum L-glutamic acid, methyl β-D-galactopyranoside, L-threo-3-methylaspartate, and histidine levels. The relative abundance of dgA-11_gut_group was significantly and negatively correlated with serum N-phenylacetylglycine level. The relative abundance of *Bacteroides* spp. was significantly and negatively correlated with serum N-phenylacetylglycine, FFA (18:3), LPE (18:1/0:0), LPE (0:0/18:1), LPC (19:2/0:0), Galacturia (19:2/0:0), Galacturia (18:1/0:0), galacturonic acid, Pro-Ile, fumaric acid, estrone, and maleic acid levels.

**Figure 8:**
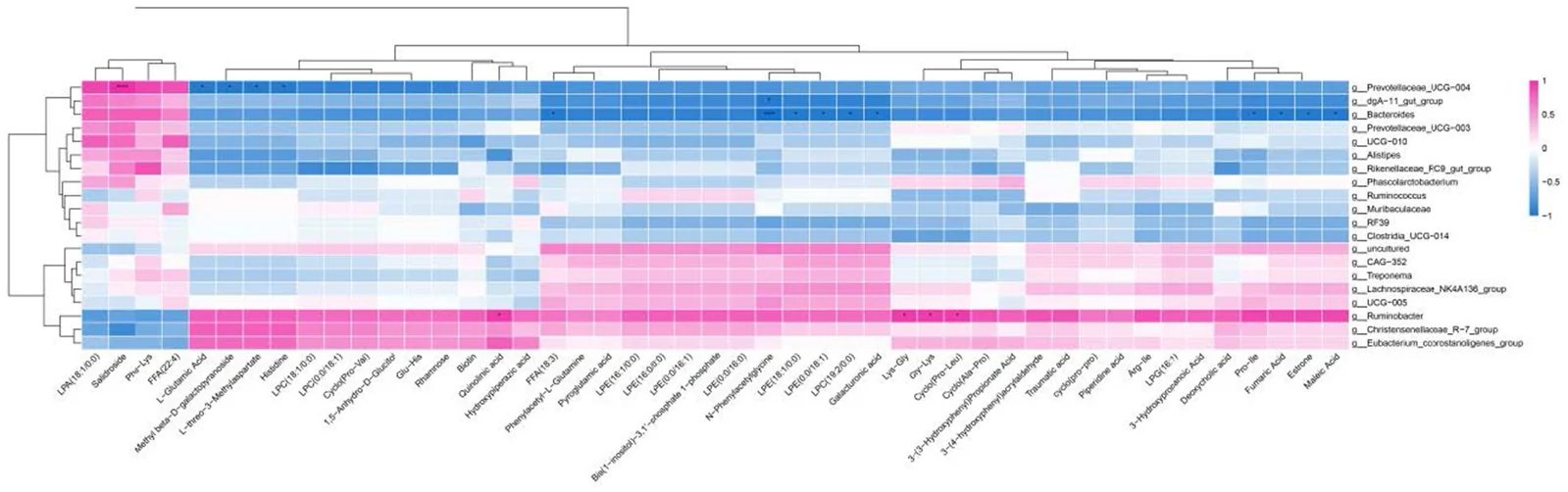
Serum differential metabolite and differential bacterial genus correlation analyses. * indicates significant correlation (*P* < 0.05) and **** indicates highly significant correlation (*P* < 0.0001). Red indicates positive correlations, whereas blue indicates negative correlations.

## 4 Discussion

During animal development, the gut microbiota forms a synergistic relationship with the host and plays an important role in nutrient absorption and metabolism, with beneficial and potentially harmful bacteria maintained in a dynamic balance (42). *Firmicutes* and Bacteroidota are two major beneficial bacterial phyla colonizing the gut. Firmicutes can effectively degrade dietary fiber, thereby promoting host digestion and nutrient absorption. Bacteroidota mainly participates in the degradation of noncellulosic plant components and can ferment plant polysaccharides to produce short-chain fatty acids, such as acetate and propionate (43, 44). In this study, dietary supplementation with wall-broken yeast powder did not significantly affect gut microbial diversity in FMD. However, the relative abundance of Firmicutes was significantly higher in the BWY group than in the CK group, whereas Bacteroidota showed a decreasing trend without statistical significance. These results suggest that wall-broken yeast powder may modulate the gut microbiota of FMD. In addition, an increase in the relative abundance of *Proteobacteria* can cause instability and dysbiosis of the host intestinal microbiota. *Proteobacteria* are facultative anaerobes that produce lipopolysaccharide, which can induce inflammatory responses and exhibit pathogenic potential. In this study, Proteobacteria were detected in the intestines of both the CK and BWY groups, which may be related to the feeding environment (45). Moreover, the short feeding period may have prevented the gut microbiota from reaching a stable state (46). At the genus level, wall-broken yeast powder increased the relative abundances of *Ruminobacter*, UCG-005, and *Bacteroides*. As ruminants rely heavily on microbial fermentation, the rumen plays a key role in cellulose degradation, nutrient utilization, and productive performance, with feed efficiency closely associated with the gut microbiota. Deepthi et al. (47) found that Kasaragod Dwarf cattle, a ruminant breed with higher feed efficiency, had a higher abundance of *Ruminobacter*. In addition, Huang et al. (48) reported that dietary supplementation with phlorotannin extracts promoted rumen fermentation in ruminants by increasing dry matter digestibility and gas production, with *Ruminobacter* playing an important role in PT-regulated rumen biohydrogenation.UCG-005 belongs to Ruminococcaceae, and can degrade dietary cellulose to produce SCFAs, which play important roles in intestinal immunomodulation, appetite and energy intake regulation, glucose homeostasis, and lipid metabolism (49, 50). The relative abundance of UCG-005 increased in diabetic nephropathy rats after treatment with San-Huang-Yi-Shen capsules. Similarly, rumen-protected glucose supplementation increased UCG-005 abundance in early-lactation dairy cows, which may help reduce gut inflammation and prevent related metabolic diseases (51, 52). Meanwhile, yeast culture alleviated rumen epithelial damage in sheep and increased the relative abundance of UCG-005, which is consistent with our findings (8). The genus *Bacteroides* has been reported to participate in nutrient metabolism and contribute to intestinal microbial stability (53). In addition, the BWY group showed a significant decrease in the abundance of *Prevotellaceae_UCG-003* compared with the CK group. This genus has been reported to be associated with intestinal dysbiosis under certain pathological conditions (54–56). In the present study, BWY supplementation was associated with changes in the abundance of several bacterial taxa, including increased relative abundances of *Ruminobacter*, UCG-005, and *Bacteroides*. These microbial alterations suggest that BWY may contribute to maintaining intestinal microbial balance and nutrient utilization capacity. However, further functional studies are required to determine whether these microbial changes directly affect host health outcomes.

Metabolomic analysis can provide a better understanding of how feeds affect animal metabolic processes, thereby helping optimize feed formulations and improve animal performance. In this study, 69 differential metabolites were identified between the CK and BWY groups, of which 63 were significantly upregulated and 6 were significantly downregulated. These metabolites were mainly enriched in amino acids and their derivatives, hormones and hormone-related compounds, glycerophospholipids, carbohydrates and their derivatives, bile acids, and organic acids and their derivatives. The metabolic pathways involved ABC transporters, glycerophospholipid metabolism, phenylalanine metabolism, carbon metabolism, and β-alanine metabolism. Histidine is an essential amino acid obtained from the diet and has antioxidant, antiinflammatory, and chelating properties. Therefore, it may play a role in neurodegenerative and age-related diseases, including Alzheimer's disease, cataracts, cancer, and atherosclerosis; as well as metabolic syndromes, including obesity, hypertension, and insulin resistance, rheumatoid arthritis; inflammatory bowel disease, including Crohn's disease and ulcerative colitis, and other diseases. Its expression levels are negatively correlated with these diseases (57, 58). Excessive zinc can induce neuronal death, whereas histidine prevents Zn^2+^ accumulation in GT1-7 cells and protects against zinc-induced neurotoxicity (59), Li et al. (60) also showed that non-esterified fatty acids induced inflammatory responses in bovine mammary epithelial cells, whereas L-histidine attenuated this inflammation by inhibiting Gab2 expression. In this study, histidine-related metabolites were upregulated in the BWY group, suggesting that wall-broken yeast powder may influence host metabolic regulation through alterations in histidine metabolism. Glutamate is a non-essential amino acid and one of the common amino acids in mammals. It is also a substrate of glutamyltransferase, a key enzyme involved in the conversion of glutamate to γ-glutamyl compounds. γ-glutamyl compounds are precursors of several important bioactive molecules, including glutathione, and are involved in antioxidant defense, detoxification, immune regulation, and other physiological processes. Therefore, glutamate may function as a bioactive compound with antioxidant, detoxifying, and immunomodulatory potential. Therefore, glutamate can be used as a functional amino acid that participates in metabolism and regulates key physiological processes, thereby participating in metabolic regulation and physiological processes (61, 62). In addition, we found that estrone levels were significantly higher in FMD supplemented with wall-broken yeast powder, suggesting that wall-broken yeast powder may increase estrogen levels and promote estrus, courtship, and reproductive behaviors in females (63). In particular, the main metabolites involved in glycerophospholipid metabolism were LPC (0:0/18:1), LPC (19:2/0:0), LPE (18:1/0:0), and LPE (16:1/0:0). Phosphatidylcholine and phosphatidylethanolamine can be catalyzed into LPC and LPE by phospholipase A1/A2, and these metabolites are negatively correlated with inflammatory markers (64). In this experiment, the levels of these metabolites increased significantly in the BWY group. These metabolic alterations suggest that BWY supplementation may influence host metabolic homeostasis by regulating lipid-related metabolites and amino acid availability. Glycerophospholipid metabolites, such as LPC and LPE, are involved in membrane remodeling, cellular signaling, and inflammatory regulation, whereas amino acids such as histidine and glutamate participate in antioxidant defense, nutrient metabolism, and immune-related physiological processes (58, 62, 65). Therefore, the changes observed in serum metabolites may reflect potential metabolic adaptations associated with intestinal microbial modulation. However, these potential physiological effects require further validation through direct measurements of immune parameters and growth performance.

Correlation analysis showed that *Prevotellaceae_UCG-004* was positively correlated with salidroside. *Prevotellaceae_UCG-004* can degrade carbohydrates to produce SCFAs, including butyrate, acetate, and propionate (66); However, although *Prevotellaceae_UCG-004* was positively correlated with salidroside, its relative abundance was lower in the BWY group than in the CK group. Shao et al. (67) investigated the effects of soybean isoflavones on ruminal microbiota in fattening goats and found that SIFs decreased the abundance of *Prevotellaceae_UCG-004*. Similarly, Belanche et al. (68) reported that the addition of PEY, consisting of turmeric, thymol, and yeast cell wall components, to neonatal ewe goat diets altered bacterial communities, possibly due to the antimicrobial properties of turmeric and thymol, thereby reducing the abundance of *Prevotellaceae_UCG-004*. Our results are consistent with these findings, suggesting that certain components or functional effects of yeast may contribute to the decreased abundance of *Prevotellaceae_UCG-004*. However, salidroside, an active compound isolated from *Rhodiola rosea*, was also downregulated. Salidroside has been reported to ameliorate non-alcoholic fatty liver disease by regulating gut microbiota, improving abnormal lipid metabolism, and reducing inflammatory responses. It can also alleviate cigarette smoke-induced lung inflammation by inhibiting the JNK/c-Jun pathway and exhibits anti-inflammatory, immunomodulatory, anticancer, and neuroprotective effects (69–71). In this study, the decrease in salidroside levels may be associated with the reduced abundance of *Prevotellaceae_UCG-004*. Furthermore, L-glutamic acid, histidine, methyl β-D-galactopyranoside, and L-threo-3-methylaspartate were negatively correlated with *Prevotellaceae_UCG-004*. Although L-glutamic acid plays important metabolic, nutritional, and signaling roles, excessive levels may damage neuronal cells, and interestingly, salidroside protected against L-glutamic acid-induced neuronal damage (72). These correlations suggest a potential association between *Prevotellaceae_UCG-004* abundance and serum metabolic alterations; however, the underlying mechanisms require further investigation.

The potential biological effects of BWY may be associated with its nutritional and functional components, including yeast-derived proteins, nucleotides, and β-glucans (15, 73). These components can provide substrates for intestinal microorganisms and regulate microbial ecological interactions (74, 75). In this study, the altered abundance of beneficial bacterial groups, such as *Ruminobacter*, UCG-005, and *Bacteroides*, may affect microbial fermentation processes and metabolite production. Meanwhile, changes in amino acid and glycerophospholipid metabolism indicate that BWY supplementation may influence host metabolic pathways related to nutrient utilization and physiological regulation. Through these interactions between microbial communities and host metabolism, BWY may potentially contribute to maintaining intestinal homeostasis (76). Nevertheless, immune parameters and growth performance were not directly evaluated in the present study, and future long-term studies are required to confirm these functional effects.

Several limitations of this study should be acknowledged. The relatively small sample size (*n* = 7 per group) was primarily constrained by the nationally protected status of forest musk deer and the limited availability of healthy captive individuals. Serum metabolomics analysis was further restricted to three randomly selected animals per group to minimize repeated handling stress. While this design balanced statistical power with animal welfare considerations, larger sample sizes would strengthen the reliability of the findings. The experimental duration also warrants caution in interpretation. A 7-d adaptation period was used to acclimate animals to the diet and housing conditions, followed by a 30-d feeding period based on practical captive management and previous dietary intervention studies in this species (64). Nevertheless, this timeframe may not capture long-term or stable shifts in gut microbiota and serum metabolome, and longer intervention periods would be necessary to assess sustained effects. Another consideration is that only adult male FMD were included. Given that gut microbiota composition and host metabolic responses are known to vary with sex, age, and physiological status, the present findings may not be generalizable to females or to juvenile and growing animals. Future work should incorporate both sexes and multiple age groups to determine whether the efficacy of BWY supplementation is sex or age-dependent. Although this dose was selected based on preliminary considerations, the absence of additional dose levels precludes identification of a dose response relationship or determination of the optimal supplementation rate. Multiple inclusion levels should be evaluated in subsequent experiments. Finally, although calculations indicated that the 15 g/kg DM addition contributed only ~0.65% additional crude protein, we cannot entirely exclude the possibility that this minor nutritional difference contributed to some of the observed effects. Studies using diets with equivalent nutrient profiles would help separate the functional contributions of yeast-derived bioactive compounds from their nutritional contributions. In summary, future studies with larger sample sizes, longer intervention periods, inclusion of both sexes and multiple age groups, multiple dose levels, and nutritionally matched diets are warranted to further validate and extend the present findings.

## 5 Conclusion

Dietary supplementation with wall-broken yeast powder altered gut microbial composition and serum metabolic profiles in captive forest musk deer, particularly affecting *Ruminobacter*, UCG-005, *Bacteroides*, and pathways related to amino acid and lipid metabolism. These findings provide preliminary evidence for the potential application of BWY in regulating intestinal microbial ecology and metabolic function in captive FMD. Long-term studies integrating growth performance, immune indicators, and functional validation are required in future research.

## Data Availability

The datasets presented in this study can be found in the NCBI Sequence Read Archive (SRA) under BioProject accession number PRJNA1538209.
