# Supplementary material for: Effects of Wall-broken yeast powder on gut microbiota and serum metabolome in captive forest musk deer (Moschus bere-zovskii)
Source: Front Vet Sci. 2026 Oct 22;13:1978027. doi: 10.3389/fvets.2026.1978027 (PMC13735546; doi:10.3389/fvets.2026.1978027)
Supplement: Supplementary file 1 [file Data_Sheet_1.docx]

Supplementary Material

# 1 Supplementary Figures and Tables

## 1.1 Supplementary Figures

**Supplementary Figure S1.** Rarefaction curves of fecal microbiota samples from CK and BWY groups based on 16S rRNA sequencing. The curves represent the relationship between sequencing reads and observed OTU numbers. Most samples showed a gradual tendency toward a plateau with increasing sequencing depth, indicating sufficient sequencing coverage.

## 1.2 Supplementary Figures

Supplementary Table S1. Comprehensive list of differential metabolites identified between CK and BWY groups based on serum metabolomics analysis.

| Index | Compounds | VIP | P-value | Fold-Change | Trend |
| --- | --- | --- | --- | --- | --- |
| MEDP0100 | 3-(4-hydroxyphenyl)acrylaldehyde | 1.62 | 0.03 | 1.42 | up |
| MEDP0658 | Ethyl-dodecanoate | 1.58 | 0.05 | 1.22 | up |
| MEDP2031 | alpha-Methyl-m-tyrosine | 1.62 | 0.03 | 1.32 | up |
| MEDP2312*134 | Gly-Lys | 1.58 | 0.04 | 1.16 | up |
| MEDP2528*234 | Cycloleucine | 1.82 | 0.01 | 1.49 | up |
| MEDP0143 | Biotin | 1.85 | 0.01 | 2.82 | up |
| MEDP0245 | Nicotinamide-N-Oxide | 1.62 | 0.02 | 0.46 | down |
| MEDP0326 | N'-Formylkynurenine | 1.89 | 0.00 | 2.32 | up |
| MEDP0331 | Subericacid | 1.71 | 0.04 | 2.44 | up |
| MEDP0611 | N-Acetyl-L-Histidine | 1.76 | 0.01 | 1.71 | up |
| MEDP1100 | 2-Aminomethylpyrimidine | 1.69 | 0.04 | 1.49 | up |
| MEDP1701*276 | LPC(0:0/18:1) | 1.82 | 0.00 | 1.76 | up |
| MEDP1835 | Methyl-p-tert-butylphenylacetate | 1.70 | 0.01 | 1.52 | up |
| MEDP1885 | Pro-Ile | 1.88 | 0.00 | 25.06 | up |
| MEDP1928 | Cyclo(Pro-Val) | 1.86 | 0.04 | 22.78 | up |
| MEDP2379 | Phe-Lys | 1.75 | 0.02 | 0.73 | down |
| MEDP2382 | O-Phospho-L-tyrosine | 1.67 | 0.02 | 1.64 | up |
| MEDP2428 | Cyclo(Ala-Pro) | 1.89 | 0.01 | 8.12 | up |
| MEDP2446 | cyclo(pro-pro) | 1.86 | 0.01 | 3.68 | up |
| MEDP2683 | GSK 4716 | 1.86 | 0.03 | 3.09 | up |
| MEDP1120 | Estrone | 1.89 | 0.04 | 13.14 | up |
| MEDP1919 | Cyclo(Pro-Leu) | 1.89 | 0.00 | 16.23 | up |
| MEDP2325 | Glu-His | 1.89 | 0.04 | 6.49 | up |
| MEDP2363 | Arg-Ile | 1.59 | 0.04 | 4.08 | up |
| MEDP2745 | LPC(19:2/0:0) | 1.82 | 0.03 | 3.20 | up |
| MEDN0109 | Hyodeoxycholic acid | 1.70 | 0.03 | 2.65 | up |
| MEDN0342 | Sebacate | 1.68 | 0.03 | 1.63 | up |
| MEDN1024*184 | Piperidine acid | 1.87 | 0.00 | 1.40 | up |
| MEDN1161 | Quinolinic acid | 1.86 | 0.01 | 1.38 | up |
| MEDN1224 | Bis(1-inositol)-3,1'-phosphate 1-phosphate | 1.63 | 0.02 | 2.46 | up |
| MEDN1532 | Galacturonic acid | 1.83 | 0.00 | 1.45 | up |
| MEDN1656 | Hydroxypiperazic acid | 1.72 | 0.01 | 1.71 | up |
| MEDN1686*018 | L-threo-3-Methylaspartate | 1.79 | 0.01 | 1.53 | up |
| MEDN1840*054 | Pinolenic acid | 1.59 | 0.04 | 1.62 | up |
| MEDN1899 | Deoxycholic acid | 1.76 | 0.01 | 1.88 | up |
| MEDN0012 | Histidine | 1.85 | 0.00 | 1.35 | up |
| MEDN0066 | Phenylacetyl-L-Glutamine | 1.84 | 0.00 | 5.94 | up |
| MEDN0406 | 3-(3-Hydroxyphenyl)Propionate Acid | 1.57 | 0.04 | 2.34 | up |
| MEDN0684 | Methyl beta-D-galactopyranoside | 1.87 | 0.02 | 1.85 | up |
| MEDN0720*037 | 2-Methylhippuric acid | 1.61 | 0.04 | 1.81 | up |
| MEDN1246 | 3-(pyrazol-1-yl)-L-alanine | 1.69 | 0.03 | 1.27 | up |
| MEDN1285*215 | LPE(0:0/16:0) | 1.80 | 0.02 | 1.62 | up |
| MEDN1287*214 | LPE(0:0/16:1) | 1.63 | 0.03 | 1.99 | up |
| MEDN1521 | Antineoplaston A10 | 1.90 | 0.01 | 4.47 | up |
| MEDN1589 | Traumatic acid | 1.68 | 0.04 | 2.98 | up |
| MEDN1608 | FFA(22:4) | 1.67 | 0.02 | 0.72 | down |
| MEDN1643 | LPA(18:1/0:0) | 1.86 | 0.02 | 0.44 | down |
| MEDN1751 | 2-Hexyldecanoic acid | 1.66 | 0.03 | 0.82 | down |
| MEDN1784 | N-Acetyl-L-tryptophanamide | 1.59 | 0.05 | 1.56 | up |
| MEDN1878 | Trolox | 1.62 | 0.04 | 2.16 | up |
| MEDN2260 | Salidroside | 1.61 | 0.03 | 0.44 | down |
| MEDN2366*218 | LPE(0:0/18:1) | 1.83 | 0.01 | 1.77 | up |
| MEDN2375 | LPG(16:1) | 1.74 | 0.01 | 2.00 | up |
| MEDN0332*005 | Maleic Acid | 1.91 | 0.01 | 9.54 | up |
| MEDN1496 | 2,6-Dimethoxybenzoic acid | 1.91 | 0.01 | 8.85 | up |
| MEDN0232*027 | Rhamnose | 1.92 | 0.00 | 10.70 | up |
| MEDN0293 | 3-Hydroxypropanoic Acid | 1.70 | 0.05 | 1.91 | up |
| MEDP2545*134 | Lys-Gly | 1.58 | 0.04 | 1.16 | up |
| MEDP0036*234 | Pyroglutamic acid | 1.82 | 0.01 | 1.49 | up |
| MEDP2697*276 | 2-Thio-PAF | 1.82 | 0.00 | 1.76 | up |
| MEDP1339*276 | LPC(18:1/0:0) | 1.82 | 0.00 | 1.76 | up |
| MEDN0011*018 | L-Glutamic Acid | 1.79 | 0.01 | 1.53 | up |
| MEDN0378*054 | FFA(18:3) | 1.59 | 0.04 | 1.62 | up |
| MEDN0061*037 | N-Phenylacetylglycine | 1.61 | 0.04 | 1.81 | up |
| MEDN0366*215 | LPE(16:0/0:0) | 1.80 | 0.02 | 1.62 | up |
| MEDN2364*214 | LPE(16:1/0:0) | 1.63 | 0.03 | 1.99 | up |
| MEDN0362*218 | LPE(18:1/0:0) | 1.83 | 0.01 | 1.77 | up |
| MEDN0490*005 | Fumaric Acid | 1.91 | 0.01 | 9.54 | up |
| MEDN0210*027 | 1,5-Anhydro-D-Glucitol | 1.92 | 0.00 | 10.70 | up |
